# Dependence, abuse, and psychosocial characteristics of patients transported to the emergency department due to overdose of over‐the‐counter drugs

**DOI:** 10.1002/pcn5.70181

**Published:** 2025-08-29

**Authors:** Saeko Kohara, Michiko Takai, Ryoko Kyan, Kenji Yamamoto, Hidehito Miyazaki, Masafumi Yoshimura, Yoshito Kamijo

**Affiliations:** ^1^ Department of Clinical Toxicology Saitama Medical University Hospital; ^2^ Department of Critical Care Medicine and Trauma National Hospital Organization Disaster Medical Center; ^3^ Department of Psychiatry Tokai University School of Medicine; ^4^ Department of Psychiatry Yokohama City University School of Medicine; ^5^ Department of Occupational Therapy, Faculty of Rehabilitation Kansai Medical University

**Keywords:** drug abuse, drug dependence, emergency ward, over‐the‐counter drugs, youth

## Abstract

**Aim:**

This study examined the conditions of drug abuse and dependence as well as the psychosocial characteristics of patients transported to an emergency department for over‐the‐counter (OTC) drug overdose.

**Methods:**

Participants were patients who presented to the emergency department due to an overdose of OTC drugs. Patients were evaluated using the Drug Abuse Screening Test‐20 (DAST‐20), Mini International Neuropsychiatric Interview (MINI), and an original questionnaire.

**Results:**

The study included 52 patients from four emergency medical facilities (20 males and 32 females) with a mean age of 23.6 years (standard deviation = 10.1). The DAST‐20 indicated that 17 patients (32.7%) experienced moderate or severe drug abuse. The average score for suicide risk on the MINI was 23.7 (median: 27). The severity of OTC drug abuse and dependence significantly correlated with drug overdose history (*r* = 0.44, *p* < 0.01), OTC drug type (antitussives and expectorants) (*r* = 0.36, *p* < 0.01), experience with OTC drug abuse (*r* = 0.53, *p* < 0.01), overdose purpose (relief of distress) (*r* = 0.41, *p* < 0.01), and overdose purpose (suicide) (*r* = −0.52, *p* < 0.01). The severity of drug abuse and dependence was best predicted by the combination of experience with OTC drug abuse, an advisor, and suicidal purpose (*R* ^2^ = 0.53, *p* < 0.001).

**Conclusion:**

It is crucial to investigate patient experiences with OTC drug abuse and their motivations and backgrounds for overdosing. Support for abuse and dependence should be provided in the early stages of treatment.

## INTRODUCTION

Emergency departments receive patients daily who attempt suicide or self‐harm. Acute drug overdose accounts for a large proportion of these cases and is most frequently associated with psychotropic medications. Although there have been many documented cases of psychotropic medication overdoses, there has been a recent increase in the number of cases related to over‐the‐counter (OTC) drugs.^1^, ^2^

Since 1998, the World Health Organization has recommended self‐medication and self‐care.^3^ In 2014, the Pharmaceutical Affairs Law in Japan underwent a comprehensive revision, resulting in easier procurement of OTC drugs not only from licensed pharmacies and drugstores but also through online channels. OTC drug overdoses have become a major public health concern within and outside Japan.^4^, ^5^ There have been numerous reports of an increase in self‐harm and suicidal behavior due to OTC drug overdose, particularly among females and adolescents.^6^ Recent reports also indicate an increasing trend in severe cases involving acetaminophen and ibuprofen.^7^, ^8^ While the choice of drugs may vary depending on the country, region, and era, it is clear that OTC drug overdose is a global problem.

Japan has also experienced escalating abuse and dependence on OTC drugs, particularly among youth.^9^ Over half of drug‐related disorders in adolescents are associated with OTC drugs as the primary substance.^10^ According to a survey conducted by Shimane et al.,^11^ the number of patients with drug‐related disorders who visited psychiatric clinics and were dependent on OTC drugs increased by approximately sixfold between 2012 and 2020. However, no studies have investigated the severity of abuse and dependence in patients who have overdosed on OTC drugs and been transported to emergency departments. The clinical and psychosocial characteristics, including suicidal tendencies, associated with OTC drug abuse and dependence remain unclear, making it impossible to take appropriate measures or intervene appropriately.

In April 2021, we conducted a multicenter survey on abuse and dependence in patients who presented to the emergency departments of four medical institutions in Japan with OTC drug overdoses. This survey was part of a project overseen by the Physical Emergency Coordination Committee of the Japanese Society of General Hospital Psychiatry. Data from this survey were utilized, and the actual conditions of abuse and dependence, as well as the psychosocial characteristics of patients presenting to an emergency department for OTC drug overdose in Japan, were examined.

## METHODS

### Participants

Participants were patients who were transported or came directly to an emergency department due to an overdose of OTC drugs between April 2021 and December 2022 and who agreed to participate in the study. An overdose was defined as the ingestion of a dose higher than the recommended dosage for the intended purpose. Patients who did not agree to participate or were deemed unable to complete the questionnaire at the discretion of an emergency physician were excluded from the study.

### Measures

The Drug Abuse Screening Test‐20 (DAST‐20) is a 20‐item, self‐administered instrument that measures the severity of problems related to drug use.^12^ Patients answer “yes” or “no” to questions based on their experiences over the past 12 months. The severity of the problem (normal, mild, moderate, or severe) and the necessary response (observation, brief counseling, outpatient treatment, or intensive treatment) are determined using the scores. The Suicide Risk section of the Mini International Neuropsychiatric Interview (MINI) was also used to assess suicidal tendencies.^13^ This section consists of six items asking about recent or lifetime experiences of suicidal ideation, plans, or attempts. A total score ≥10 indicated a high suicide risk.

Additionally, an original questionnaire was used to collect information on age, sex, marital status, living environment, medical history, details of OTC drugs, reasons for overdose, physical complications, length of hospital stay, outcome, history of overdose, abuse of OTC drugs (source of information, start date, purpose of abuse, and regular use), and social resources for consultation (Figure S1).

### Procedure

A description of the study was provided to participants who were transported or presented directly to the emergency department for an OTC drug overdose. The informed consent form included a description of the study, its intent and methodology, the approximate time required for participation, participants’ rights to withdraw and refuse, and the maintenance of confidentiality. Participants who agreed to sign the informed consent form were asked to complete the DAST‐20, MINI, and the original questionnaire. All questionnaires were collected and analyzed at the Department of Clinical Toxicology, Saitama Medical University Hospital.

### Outcomes

The primary outcome was the identification of psychosocial and clinical factors associated with the severity of OTC drug abuse and dependence, as measured by the DAST‐20.

### Data analyses

The demographic characteristics and scale scores of patients experiencing OTC drug overdoses were calculated. Spearman's rank correlation coefficients were calculated across all variables to determine the relationships between the severity of abuse and dependence on OTC drugs, suicidal tendencies, and other clinical and psychosocial characteristics. Stepwise multiple regression analysis was conducted to examine the predictive factors for the severity of abuse and dependence on OTC drugs. The predictive variables included income status, types of OTC drugs (antitussives and expectorants), experience with OTC drug abuse, purpose of OTC drug overdose (relief of mental distress or suicide), and presence of advisors; the criterion variable was DAST‐20 scores. Six explanatory variables that yielded a model with a high degree of goodness‐of‐fit were selected using *R*² as the evaluation criterion. IBM® SPSS® version 22.0 was used for statistical analysis, with an *α* level of 0.05 set as the threshold for statistical significance.

## RESULTS

### Demographic characteristics and scale scores

Fifty‐two patients from four emergency medical facilities were included in this study. Of these patients, 20 (38.5%) were male, and 32 (61.5%) were female, with a mean age of 23.6 years (standard deviation: 10.1). The demographic characteristics of the patients and the means and standard deviations of the DAST‐20 and MINI scores are presented in Table 1. The combined percentage of patients in their teens and 20s accounted for 80.8% of the total, indicating that patients who overdose on OTC drugs are primarily young. More than 80% of patients were students or worked full‐time. Classification revealed that 22 (29.7%) of the 74 types of medicine involved OTC drugs with acetaminophen or ibuprofen as the main ingredient; this was followed by hypnotic sedatives containing antihistamines (*n* = 17; 23.0%) and cold medications (*n* = 13; 17.6%).

**Table 1:** Demographic characteristics and DAST‐20 and M.I.N.I. scores of the participants (*N *= 52).

	Men (*n *= 20)	Women (*n* = 32)
Age in years, mean (SD)	26.3 (9.6)	21.8 (10.2)
Marital status, *n* (%)		
Single	15 (75.0)	25 (78.1)
Married	2 (10.0)	6 (18.8)
Divorced	2 (10.0)	1 (3.1)
Unknown	1 (5.0)	0
Employment status, *n* (%)		
Employed	10 (50.0)	8 (25.0)
Part‐time	1 (5.0)	1 (3.1)
Student	6 (30.0)	20 (62.5)
Unemployed	2 (10.0)	2 (6.3)
Not specified	1 (5.0)	1 (3.1)
Lives with family or partner, *n* (%)	15 (75.0)	28 (87.5)
History of psychiatric treatment, *n* (%)	10 (50.0)	17 (53.1)
History of drug overdose, *n* (%)	9 (45.0)	20 (62.5)
Experience with OTC drug abuse, *n* (%)	3 (15.9)	13 (40.6)
Types of OTC drug (Total 74 cases), *n* (%)		
Antipyretic analgesics	7 (26.9)	15 (31.3)
Hypnotic Sedatives (included antihistamines)	8 (30.8)	9 (18.8)
Caffeinated supplements	4 (15.4)	5 (10.4)
Cold medicines	3 (11.5)	10 (20.8)
Chinese herbal preparations	2 (7.7)	1 (2.1)
Gastrointestinal medications	0	1 (2.1)
Antitussives and Expectorants	2 (7.7)	7 (14.6)
Purpose of OTC drug overdose (Total 69 cases), *n* (%)		
Suicide	18 (75.0)	25 (55.6)
Relief of mental distress	3 (12.5)	10 (22.2)
Raise mood and motivation	1 (4.2)	3 (6.7)
Other purposes	2 (8.3)	7 (15.5)
Sources of information about OTC drug overdoses, *n* (%)		
SNS	1 (4.8)	11 (32.4)
Friends and/or acquaintances	2 (9.5)	2 (5.9)
Internet search	10 (35.3)	12 (35.3)
Books and/or Magazines	4 (19.0)	0
Other sources	4 (19.0)	9 (26.5)
Advisors (Total 75 cases), *n* (%)		
Family	6 (30.0)	14 (43.8)
Friends/acquaintances	3 (15.0)	7 (21.9)
Partner	1 (5.0)	4 (12.5)
Medical professional (psychiatry/psychosomatic)	3 (15.0)	8 (25.0)
Psychologist/counselor	1 (5.0)	4 (12.5)
Medical professional (physical health)	1 (5.0)	0
Teacher/school nurse	0	1 (3.1)
Others	2 (10.0)	4 (12.5)
None	9 (45.0)	7 (21.9)
DAST‐20 score, mean (SD)	4.0 (1.5)	5.4 (2.8)
Substantial (scores 11–15), *n* (%)	0	3 (9.4)
Moderate (scores 6–10), *n* (%)	4 (20.0)	10 (31.3)
Low (scores 1–5), *n* (%)	16 (80.0)	19 (59.4)
M.I.N.I. score, mean (SD)	23.3 (10.5)	23.9 (10.2)

Abbreviations: DAST‐20, The Drug Abuse Screening Test; M.I.N.I., Mini International Neuropsychiatric Interview; OTC, over‐the‐counter; SNS, Social Networking Service.

The DAST‐20 results indicated that 35 patients (67.3%) had low‐level problems related to OTC drug abuse, whereas 17 patients (32.7%) had moderate or more severe problems requiring outpatient or intensive treatment. The average score for the suicide risk section of the MINI was 23.7 (median, 27), indicating that patients were in a very high‐risk psychological state for suicide.

### Relationships between DAST‐20 Score, MINI score, and other clinical and psychosocial characteristics

Spearman's rank correlation coefficients were calculated for all variables to determine the relationship between the severity of abuse and dependence on OTC drugs, suicidal tendencies, and other clinical and psychosocial characteristics (Table 2). The DAST‐20 scores were significantly correlated with a history of drug overdose, sources of information about OTC drug overdoses (friends and/or acquaintances), types of OTC drugs (antitussives and expectorants), experience with OTC drug abuse, purpose of OTC drug overdose (relief of mental distress and increased mood and motivation), and the presence of advisors (people or organizations that patients consult with regarding their concerns, such as family members, friends, acquaintances, and medical institutions.). A negative correlation was observed between DAST‐20 scores and the purpose of OTC drug overdose (suicide). The MINI scores demonstrated a significant correlation with sources of information about OTC drug overdoses (internet search) and the purpose of OTC drug overdose (suicide), while exhibiting a negative correlation with income status.

**Table 2:** Spearman's rank correlation coefficients for DAST‐20 and M.I.N.I. scores.

	DAST‐20	M.I.N.I.
Income status	−0.02	−0.32*
Types of OTC drug	0.36**	−0.21
Antitussives and expectorants		
History of drug overdose	0.44**	−0.11
Sources of information about OTC drug overdoses	0.27*	0.06
Friends and/or acquaintances		
Internet search	−0.15	0.40**
Experience with OTC drug abuse	0.53**	−0.04
Purpose of OTC drug overdose	−0.52**	0.29*
Suicide		
Relief of mental distress	0.41**	−0.05
Raise mood and motivation	0.34*	0.05
Presence of advisor	0.34*	−0.02

*Note*: DAST‐20, The Drug Abuse Screening Test; M.I.N.I., Mini International Neuropsychiatric Interview; OTC, over‐the‐counter.

* *p* < 0.05

** *p *< 0.01 (two‐tailed).

Stepwise multiple regression analysis revealed that experience with OTC drug abuse was the strongest predictor of the severity of abuse and dependence on OTC drugs (*F* (1, 50) = 23.31, *p* < 0.001), accounting for 32% of the variance in DAST‐20 scores. The severity of abuse and dependence on OTC drugs was best predicted by the combination of experience with OTC drug abuse, presence of an advisor, and purpose of OTC drug overdose (suicide) (*F* (3, 48) = 17.65, *p* < 0.001), accounting for 53% of the variance in DAST‐20 scores (Table 3).

**Table 3:** Stepwise multiple regression for DAST‐20 scores.

Model		B	SE	*β*	*F*	R^2^
1	Experience with OTC drug abuse	3.00	0.62	0.56	23.31**	0.32
2	Experience with OTC drug abuse	3.19	0.56	0.60	20.89**	0.46
	Presence of advisor	2.01	0.56	0.38		
3	Experience with OTC drug abuse	2.81	0.55	0.53	17.65**	0.53
	Presence of advisor	1.70	0.55	0.32		
	Purpose of OTC drug overdose: Suicide	‐1.74	0.68	‐0.27		

Abbreviations: DAST‐20: The Drug Abuse Screening Test; OTC, over‐the‐counter.

** *p* ≤ 0.01 (two‐tailed).

## DISCUSSION

In this study, a multicenter questionnaire survey was conducted with patients who presented to the emergency department for OTC drug overdose, and the psychosocial characteristics related to abuse and dependence were examined. To our knowledge, no previous studies have investigated the issue of abuse and dependence in patients who have overdosed on OTC drugs and have been admitted to the emergency department. Abuse and dependence were related to prior experience with abusing OTC drugs, the presence of an advisor, and the purpose of the overdose being something other than suicide (“relief of mental distress” or “raise mood and motivation,” etc.). These findings underscore the importance of providing effective support and interventions to patients in emergency medical settings.

A significant proportion of patients who were transported to the hospital for an overdose of OTC drugs were in their teens or 20s. This finding is consistent with a previous report showing that approximately 40% of patients aged <20 years who visited a psychiatric clinic with drug‐related disorders had a history of OTC drug abuse, indicating that the number of cases of OTC drug overdose, particularly among young people, is increasing.^11^ The most common employment status among patients was students, followed by those with full‐time employment. Past reports have indicated that the backgrounds of patients who overdose are often “unemployed” or “isolated/lonely”^14^. Conversely, there are conflicting reports that people who abuse OTC drugs often have high levels of education, no history of truancy or delinquency, and a lifestyle background that is no different from that of young people who do not overdose.^15^ Even outside Japan, reports indicate that patients who overdose on　pharmaceutical drugs tend to be higher educated and higher socioeconomic strata.^16^ This finding indicates that young people who have been leading seemingly problem‐free lives as students or full‐time workers may use OTC drugs to manage mental health issues.

Correlation analysis identified an association between the severity of abuse and dependence on OTC drugs, history of drug overdose, sources of information about OTC drug overdoses (friends or acquaintances), the purpose of OTC drug overdose (relief of mental distress or raised mood, and motivation), and the presence of advisors. Previous studies have reported that the probability of reusing OTC drugs is high and that OTC drug dependence is severe.^17^, ^18^ It is possible that easily accessible OTC drugs can induce drug dependency. In a report by Kyan et al.,^19^ patients who regularly took OTC drugs were significantly more likely to actively seek drug‐related information on the Internet and disseminate it on their social networks. Disclosure of personal drug use behaviors online to connect with peers under similar circumstances and sharing information to gain acceptance and approval from peers may contribute to the prevalence of OTC drug overdoses among many adolescents.^19^ This study's results also indicated that an overdose of OTC drugs with friends or acquaintances to relieve mental distress or raise mood and motivation may be repeated, leading to abuse and dependence.

The presence of advisors was found to be associated with the severity of OTC drug abuse and dependence. Among individuals who reported having an advisor, approximately half described them as close relationships, including family members, friends, acquaintances, or partners. This finding suggests that social connections function as a protective factor, enabling individuals to maintain a certain level of stability in daily life, such as attending school or work. However, the absence of appropriate medical care or support services may lead to prolonged OTC drug abuse, potentially exacerbating the severity of abuse and dependence. Additionally, approximately 20% of the participants who reported having an advisor identified mental health professionals (psychiatrists, psychologists, or counselors) as their advisors. Matsumoto^20^ reported that 30%–70% of individuals with substance dependence have comorbid psychiatric disorders, with many experiencing these psychiatric conditions before developing substance dependence. This suggests that the distress caused by these psychiatric disorders significantly impacts the use of substances such as OTC drugs.

We initially expected that the presence of advisors would have a preventive or supportive effect on OTC drug abuse and dependence; however, the results of this study showed the opposite effect. This may be due to the instability of the model resulting from multivariate analysis conducted on a small number of cases. To validate the model, further studies with a larger sample size are required.

The severity of abuse and dependence on OTC drugs was related to the type of OTC drug (antitussive or expectorant). The primary ingredient in Japanese antitussive and expectorant drugs is dextromethorphan, which has been available as an OTC drug since 2021. Dextromethorphan has been identified as a substance with a high potential for abuse and dependence, and there is a risk of relapses associated with its use.^21^ This substance was recently launched and is currently not subject to sales restrictions; however, we consider it a substance that requires further research regarding its potential dangers. This study did not examine the relationship between the number of tablets of OTC drugs taken in overdose, the number of drug types (polypharmacy), and the severity of OTC drug abuse and dependence or suicide risk. Previous studies have reported that the total number of tablets taken is associated with the need for psychiatric hospitalization and that poly‐substance use is a risk factor for overdose in young people.^22^, ^23^ Therefore, it is also possible that the number of tablets or types of drugs taken may be associated with the severity of abuse or dependence on OTC drugs. This should be investigated in future studies.

There was no association between the severity of abuse, dependence on OTC drugs, and suicidal tendencies. Suicidal tendencies were related to the fact that the source of information regarding OTC drug overdose was an “Internet search” and that the purpose of OTC drug overdose was “suicide.” In other words, patients who attempt suicide using OTC drugs after searching for information about an overdose on the internet may be at a higher risk of suicide. Given the relatively low mortality rate associated with overdoses and the ambiguity surrounding suicidal ideation, the risk of subsequent suicide tends to be underestimated in emergency medical settings.^24^, ^25^ A history of attempted suicide has been identified as a risk factor for subsequent suicide.^26^, ^27^ In a 2024 report by Matsumoto et al.,^28^ approximately 60% of patients with drug‐related mental disorders caused by OTC drugs exhibited self‐injurious behavior or attempted suicide within a year, which was a higher rate than other causes. Based on these results, it is necessary to acknowledge cases that exhibit high suicidal tendencies among individuals who have overdosed on OTC drugs; a comprehensive medical interview must be conducted, and psychiatric treatment and other forms of support must be implemented.

Stepwise multiple regression showed that experience with OTC drug abuse was the strongest predictor of the severity of abuse and dependence. Furthermore, experience with OTC drug abuse, the presence of an advisor, and the purpose of the overdose (relief of mental distress or raising mood and motivation) other than suicide explained 53% of the variance in the DAST‐20 scores. These findings suggest that dependence may develop because of repeated overdoses of OTC drugs. Patients may also use OTC drugs to foster social connections, alleviate psychological distress, and enhance their mood and motivation. The cessation of OTC drug use may not adequately address the underlying issues that participate in these behaviors. In emergency medical settings, where resources are often limited and time is essential, it is important to investigate patients’ experiences of OTC drug abuse and the underlying motivations behind their overdoses. The implementation of this approach allows patients to be informed that resolution of pain and difficulties can be achieved without resorting to unhealthy coping behaviors such as overdosing on OTC drugs. This approach enables the provision of support for substance abuse and dependence at an early stage of the treatment process. To respond to the diverse backgrounds of patients, it is imperative to prevent the recurrence of overdoses by providing support through inter‐professional collaboration. Moreover, even in cases of an OTC drug overdose, there are cases in which an individual is in a psychological state with a high risk of suicide. In such cases, it is necessary to provide sufficient psychiatric interventions to address the underlying suicidal tendencies.

This study had certain limitations. Only four emergency departments and 52 cases were examined, resulting in a relatively small sample size. A multiple regression analysis was performed using six explanatory variables, raising concerns about overfitting and reduced statistical power. There is also the possibility of selection bias, as patients who cooperated with the study may not be representative of the entire population. In future research, we anticipate the formulation of universally applicable and effective measures and support through the augmentation of facilities and cases.

In conclusion, we examined drug dependence and abuse factors along with psychosocial characteristics of patients presenting to emergency departments for OTC drug overdoses. It is important to investigate patients’ experiences with OTC drug abuse and the underlying motivations for overdosing, and it is crucial to provide support for abuse and dependence at the early stage of the treatment process.

## AUTHOR CONTRIBUTIONS

Saeko Kohara and Michiko Takai designed the study, developed the main conceptual ideas, and drafted the manuscript with the assistance of Ryoko Kyan, Kenji Yamamoto, Hidehito Miyazaki, Masafumi Yoshimura, and Yoshito Kamijo. Saeko Kohara, Michiko Takai, Ryoko Kyan, Kenji Yamamoto, Hidehito Miyazaki, Masafumi Yoshimura, and Yoshito Kamijo collected the data and assisted in interpreting the results.

## CONFLICT OF INTEREST STATEMENT

The authors declare no conflict of interest.

## ETHICS APPROVAL STATEMENT

This study complied with all the relevant national regulations and institutional policies related to research involving human subjects and was conducted in accordance with the tenets of the Helsinki Declaration. The study protocol was approved by the Institutional Review Board of the Ethics Committee of Saitama Medical University Hospital (2025‐015).

## PATIENT CONSENT STATEMENT

All study participants or their legal guardians provided written informed consent before study enrollment.

## CLINICAL TRIAL REGISTRATION

N/A.

## Supporting information

Supporting information.

## Data Availability

The data supporting the findings of this study are subject to restrictions. The use of data for secondary purposes was contingent on the approval of the Physical Emergency Coordination Committee of the Japanese Society of General Hospital Psychiatry. Requests for access to these data sets should be directed to the corresponding author.
