# Supplementary material for: Dependence, abuse, and psychosocial characteristics of patients transported to the emergency department due to overdose of over‐the‐counter drugs
Source: PCN Rep. 2025 Aug 29;4(3):e70181. doi: 10.1002/pcn5.70181 (PMC12397076; doi:10.1002/pcn5.70181)
Supplement: Supplementary file 1 — Supporting information. [file PCN5-4-e70181-s001.docx]

**Fig S1: Questionnaire for Patients with Overdose of Over-the-Counter (OTC) Medications**

**Basic Information**

- Age: [ 　　] years / [　　 ] age group 　□Unknown
- Gender: □Male □Female
- **Marital Status:** □Married ( ○ Living together ○ Separated ) □Unmarried □Divorced □Widowed □ Other (　　　　 )　□Unknown
- **Employment Status:** □ Employed ( ○ Self-employed/Family business　　　　　 ○ Employed by others ) □Unemployed ( ○ Jobless ○ Student ○ Homemaker 　○ Pension/Unemployment benefits recipient ○ Other ) □Unknown
- **Cohabitants** :□Yes (specify who:　　　　 ) □No □Unknown
- **Financial Source:** □Personal income □ Family support □ Pension 　　　　□Disability pension □Savings □ Public assistance □Other (　　　　 )

**Emergency Department Visit**

- Date: 20[　　 ]/[　　 ]/[　　 ]
- Mode of arrival: □Ambulance □Walk-in □Other (　　　　)

**Details of Overdosed OTC Medications** (Please include any non-OTC drugs taken in overdose as well)：

**Background Factors Contributing to Overdose**

□Family issues □Health issues □Financial/lifestyle issues □Work-related issues □Relationship issues □School-related issues □Other 　　　　　　　　　　　　　　　　　　　　□ Unknown 　　　　　　　　　　　　　　　　　　　　　　　　　　　　　　　　　　　　　　　　　　　(Please describe the background circumstances　indetail:　　　　　　　　　　　　　　　　　　　　　　　　　　　　　　　　　　　　）

**Physical Complications Resulting from Overdose**

□Consciousness disturbance □Cardiac arrest □Shock □Respiratory failure

□Circulatory failure □Toxic symptoms □CNS symptoms 　□Other (　　　　　　 )

**Suicide Attempt Classification**

□Suicide attempt (intentional self-harm not resulting in death)

□Self-injury (intentional physical harm) □Other (　　　　　 ) □Unknown

**Medical History**

- Physical illness: □Yes (diagnosis: 　) □No □Unknown
- Psychiatric illness: □Yes (diagnosis:　　　 ) □No 　□Unknown

**History of Overdose**

□ Yes (Number of times:　　　　　 ) (Most recent: 　　　　　) □No □Unknown

**History of Suicide Attempts** (Including overdose)

□ Yes (Number of times:　　　　　 ) (Most recent:　　　　　 ) □No □Unknown

**Source of Information about OTC Drugs**

□Social media (specify: 　　　) □Partner

□Friends/Acquaintances □Other (specify:　　　 )

**Habitual Use of OTC Drugs for Non-Therapeutic Purposes**

□Yes (What:　　　 ) (Frequency/amount: 　　　) □No □Other (specify:　　　 )

**Experience Using OTC Drugs for Non-Therapeutic Purposes**

□ No □Yes (Since around age:　　　 ) □ Other (specify:　　　 ) □Unknown

**Reasons for Using OTC Drugs for Non-Therapeutic Purposes**

□Recommended or influenced by others □Recreational □To relieve unpleasant

feelings □To improve mood or motivation □Other (specify: 　　　) □Unknown

**Consultation Partners/Agencies at Time of Emergency Visit**

□None □Family ( ○ Spouse ○ Child ○ Parent ○ Sibling ○ Other ) □Relatives 　　　□ Friends/Acquaintances □Partner □Medical professional (psychiatry/psychosomatic) □Psychologist/Counselor □Medical professional (physical health) □Teacher/School nurse □Other (specify:　　 )

□None □Medical institution (psychiatry/psychosomatic) □Medical institution (physical health) □Workplace/school health center □Ward office ( ○ Welfare division ○ Child and family division ○ Other ) □Community general support center □Disability support center □Child consultation center □Child and family support center □Mental health and welfare center □Legal consultation □Telephone counseling center □Other (specify:　　　 )

**Discharge Date from Emergency Medical Facility**

- Date: 20[ 　　]/[ 　　]/[　　 ]

**Outcome at Emergency Facility**

□Death ( ○ Declared dead in outpatient ○ Died during hospitalization )

□ Discharged (Referred to: ○ Family doctor (psychiatry/psychosomatic) ○ Family doctor (other))

□Referred ( ○ Local institution: (specify: ) )

□Transferred within hospital ( ○ Psychiatric dept. ○ Other dept. )

□Transferred to another hospital ( ○ General hospital psychiatry ○ Psychiatric hospital ○ Other dept. in general hospital ○ Other )
